# Soluble OX40L is associated with presence of autoantibodies in early rheumatoid arthritis

**DOI:** 10.1186/s13075-014-0474-4

**Published:** 2014-10-30

**Authors:** Julie K Laustsen, Tue K Rasmussen, Kristian Stengaard-Pedersen, Kim Hørslev-Petersen, Merete L Hetland, Mikkel Østergaard, Peter Junker, Malene Hvid, Bent Deleuran

**Affiliations:** Department of Biomedicine, Aarhus University, Wilhelm Meyers Allé 4, 8000 Aarhus C, Denmark; Department of Rheumatology, Aarhus University Hospital, Aarhus C, Denmark; Department of Clinical Medicine, Aarhus University, Aarhus C, Denmark; King Christian 10th Hospital for the Rheumatic Disease, University of Southern Denmark, Odense M, Denmark; Copenhagen Center for Arthritis Research, Center for Rheumatology and Spine Diseases, Glostrup Hospital, Copenhagen, Denmark; Department of Clinical Medicine, Faculty of Health and Medical Sciences, University of Copenhagen, Copenhagen, Denmark; Odense University Hospital, Odense, Denmark

## Abstract

**Introduction:**

OX40 and its ligand OX40L are key components in the generation of adaptive memory response and provide necessary co-stimulatory signals for activated effector T cells. Here we investigate the dual roles of the membrane and soluble (s) forms of OX40 and OX40L in plasma and synovial fluid and their association with autoantibodies and disease activity in rheumatoid arthritis (RA).

**Methods:**

Soluble OX40 and sOX40L plasma levels were measured in treatment-naïve early RA patients (eRA) at baseline and after 3, 6, and 12 months of treatment with methotrexate and adalimumab (n = 39) and with methotrexate alone (n = 37). Adalimumab was discontinued after the first year, and patients were followed for additional 12 months. For comparison, sOX40 and sOX40L were measured in patients with chronic RA (cRA, n = 15) and healthy volunteers (HV, n = 34). Membrane-bound OX40 and OX40L expression on T cells, B cells and monocytes were quantified.

**Results:**

Soluble OX40 plasma levels of eRA patients were not different at the time of treatment initiation, but were significantly higher after 12 months of treatment, compared with HV or cRA patients. Soluble OX40L was significantly elevated throughout the first 12 months of treatment compared with HVs and patients with cRA. Adalimumab treatment did not influence sOX40 or sOX40L plasma levels. At baseline, sOX40L levels were strongly associated with the presence of anti-citrullinated protein antibodies (ACPA) (*P* <0.001) and IgM-RF (*P* <0.0001). The sOX40/sOX40L ratio was decreased in eRA, and a low ratio at the time of adalimumab discontinuation was associated with increased DAS28CRP and risk of flare the following year. T cells in the synovial fluid had the highest expression of OX40, while monocytes and B cells were the main expressers of OX40L in the joint.

**Conclusions:**

Plasma levels of sOX40 and sOX40L were increased in eRA and sOX40L was correlated with ACPA and IgM-RF. Further, expression of membrane-bound OX40 and OX40L was increased in eRA and cRA. Combined, these findings could reflect that increased activity in the OX40 systems facilitate to drive disease activity and autoantibody production in RA.

**Trial registration:**

Clincaltrials.gov NCT00660647, 10 April 2008.

**Electronic supplementary material:**

The online version of this article (doi:10.1186/s13075-014-0474-4) contains supplementary material, which is available to authorized users.

## Introduction

Rheumatoid arthritis (RA) is a chronic autoimmune disease affecting about 0.8% of the adult population. It is characterized by synovitis and progressive destruction of the joints accompanied by multiple systemic symptoms. Autoantibodies occur in 60 to 80% of patients, suggesting a pivotal role for adaptive immune responses in the pathogenesis [1]. This is supported by the presence of increased amounts of CD4 + CD45RO + T cells in the RA synovium.

Several members of the TNF superfamily play an important role for the generation of an optimal memory response; among these are OX40 and its ligand, OX40L [2-5]. OX40 is transiently induced on T cells upon antigen activation, while OX40L is expressed by a variety of cells, most abundantly on antigen-presenting cells (APCs) [6-9].

OX40 provides a co-stimulatory signal to activated effector T cells and is crucial for the generation of memory T cells and hence for the persistence of immunity [9]. The generation of memory T cells is achieved through the NF-κB pathway by induction of anti-apoptotic factors [3]. The importance of the OX40/OX40L axis in memory generation and autoimmunity has been demonstrated in several animal studies where OX40- or OX40L-deficient mice have been shown to have an impaired memory response [8,10,11]. In addition to T cells, a recent study using a graft versus host model supports the role of OX40 in B cell activation. Here, OX40 stimulation induced production of donor-reactive alloantibodies in the absence of CD40 [12].

The TNF superfamily is known to induce bidirectional signals and this also applies to OX40/OX40L [13]. In addition to serving as a ligand, OX40L is a counter receptor, which initiates reverse signals in the cell and regulates cytokine production and IgG class switch [14]. In accordance with this, the OX40/OX40L binding axis assumes an important role in sustaining an ongoing memory-prone immune response, and it is believed to be important in the pathogenesis of autoimmune diseases like RA. In support of this, animal studies demonstrate the presence of OX40 and OX40L in synovial tissue and reveal that endogenous OX40L plays a pro-inflammatory role in collagen II-induced arthritis in mice as administration of anti-OX40L mAb ameliorates the disease severity [15,16]. Besides their membrane-bound isoforms, OX40 and OX40L are both present in soluble forms (sOX40 and sOX40L) that have previously been linked to autoimmune diseases [2,4]. The bioactive nature of these soluble forms is supported by reports that sOX40 is antagonistic to the membrane-bound form because it inhibits the inflammatory response and thus, mimics the T regulatory (T-reg) function [6,8].

Based on the above, we hypothesize that RA patients show signs of activity in the OX40 system. To investigate this, we measure the plasma levels of soluble OX40 and OX40L in a closely monitored cohort of early RA patients and compare with chronic RA patients and healthy volunteers, and examine the association between these soluble isoforms, autoantibodies and clinical disease parameters. We further measure the expression levels of the membrane-bound isoforms on cells from peripheral blood and synovial fluid.

## Materials and methods

### Patient samples

A sample set of ethylenediaminetetraacetic acid (EDTA)-stabilized plasma samples was obtained from a Danish randomized controlled trial (RCT), optimized treatment algorithm in early rheumatoid arthritis (OPERA) (n = 76), which included RA patients with a disease duration of less than 6 months [12]. Samples were selected in a blinded, non-stratified manner from a longitudinal set of steroid- and disease-modifying anti-rheumatic drug (DMARD)-naïve RA patients (eRA) (n = 76) from whom samples were obtained at baseline (time of diagnosis) and after 3, 6, and 12 months of treatment. All patients received methotrexate (MTX) in combination with either adalimumab (ADA, n = 39) or placebo (PLA, n = 37). If patients presented with swollen joints during the first year, they were treated with intra-articular triamcinolone injections. Biochemical and clinical diseases parameters were measured and collected at baseline, and at 3 months and 12 months of treatment (Table 1). IgM rheumatoid factor (IgM-RF) and anti-citrullinated peptide antibody (ACPA) were measured by ELISA, the latter by using second-generation cyclic citrullinated peptide as antigen (Phadia, Uppsala, Sweden). After the first year of treatment, adalimumab was discontinued and patients were followed for disease activity for another 12 months. In this study, we defined a flare as an increased score for CRP-based disease activity in 28 joints (DAS28CRP) >1.2 if DAS28CRP <3.2 or >0.6 if DAS28CRP ≥3.2 according to van der Maas *et al*. [17]. EDTA plasma samples from healthy volunteers (HV, n = 34) matched for age and gender with the OPERA patients were obtained from blood donors at the blood bank, Aarhus University Hospital.

**Table 1:** **Characteristics of patients and controls**

**Characteristics**	**Early rheumatoid arthritis PLA (n = 37)**	**Early rheumatoid arthritis ADA (n = 39)**	**Chronic rheumatoid arthritis (n = 15)**	**Healthy volunteers (n = 34)**
Age, years	54 (37 to 65)	56 (38 to 62)	56 (45 to 64)	54 (38 to 62)
Gender, % female	78	64	80	67
Disease duration at inclusion, months	2.7 (1.4 to 4.3)	2.9 (1.6 to 4.4)	234 (120 to 342)	NA
**Disease scores**				
DAS28 score, baseline	5.6 (4.9 to 5.8)	5.4 (4.8 to 6.4)	3.0 (2.2 to 3.9)	NA
DAS28 score, 3 months	2.3 (1.9 to 2.9)	2.0 (1.8 to 2.7)	NA	NA
DAS28 score, 12 months	2.2 (1.9 to 3.4)	1.9 (1.8 to 2.5)	NA	NA
Remission, 12 months, %	56	52	NA	NA
HAQ score, baseline, range 0 to 3	0.9 (0.6 to 1.3)	1.1 (0.8 to 1.5)	1.0 (0.2 to 2.0)	NA
HAQ score, 3 months, range 0 to 3	0.1 (0.0 to 0.6)	0.2 (0.0 to 0.38)	NA	NA
HAQ score, 12 months, range 0 to 3	0.1 (0 to 0.5)	0.0 (0.0 to 0.4)	NA	NA
**Treatment dose**				
MTX, 12 months, mg/week	20.0 (15.5 to 20.0)	20.0 (18.8 to 20.0)	NA	NA
Cumulative dose of intra-articular betamethasone, 12 months, ml	8.0 (5.0 to 12.5)	5.0 (3.0 to 10.0)	NA	NA
**Laboratory findings at baseline**				
IgM-RF-positive, %	84	67	74	NA
ACPA-positive, %	81	56	74	NA

Results are presented as median (SD) or percent. Statistical difference in anti-citrullinated protein antibody (ACPA)-positive in the patient group receiving placebo and methotrexate (PLA) and the patient group receiving adalimumab and methotrexate (ADA) at baseline (*P* <0.05). MTX, methotrexate; ADA, adalimumab; CRP, C-reactive protein; DAS28, disease activity score in 28 joints; DAS28CRP, CRP-based DAS28; Remission, American College of Rheumatology/European League Against Rheumatism remission (Boolean, 28 joints); HAQ, health assessment questionnaire; IgM-RF, IgM rheumatoid factor; NA, not applicable or not available.

A cross-sectional sample set was collected from chronic RA (cRA) patients (n = 15) including peripheral blood mononuclear cells (PBMC) with paired synovial fluid mononuclear cells (SFMC) from the outpatient clinic at Aarhus University Hospital. All cRA patients had disease duration of more than 8 years. PBMCs from HV served as a control group (HV, n = 10). In comparison we collected a cross-sectional sample set of PBMC from eRA patients with disease duration less than 6 months (n = 5) from the outpatient clinic at Aarhus University Hospital.

PBMC and SFMC from EDTA-stabilized whole blood and synovial fluid, respectively, were isolated by density gradient centrifugation using Ficoll-Paque PLUS and stored at −150°C until use. Plasma was stored at −80°C until time of analysis.

### Ethics

The studies were approved by the Danish Medical Agency (2612-3393), the Danish Data Protection Agency (2007-41-0072 and 2011-41-6863), and the Regional Ethics Committee (VEK-20070008, 1-10-72-291-12 and 1-10-72-189-13). All patients gave written consent to participate in the study in accordance with the Declaration of Helsinki and carried out according to the principles of the International Conference on Harmonization guidelines for good clinical practice (GCP, 1996 revision).

### ELISA

Plasma levels of sOX40 were quantified using a sOX40 ELISA kit (catalog number BMS296, eBioscience, San Diego, CA, USA) in accordance with the manufacturer’s description with the following modifications: samples were diluted in sample diluent supplemented with human, murine and bovine IgG (all from Jackson Immunoresearch, West Grove, PA, USA) added in excess to ensure pre-aggregation of heterophilic antibodies in the samples [18]; samples and standards were added to the wells and incubated overnight at 4°C; after adding biotin-conjugated detection antibody, the plates were incubated with ELAST enhancement kit (PerkinElmer, Waltham, MA, USA) and washed seven times before adding tetramethylbenzidine (TMB). All samples were analyzed in duplicates using the average optical density (OD) values to calculate concentrations (minimum detection level (cutoff) 5.97 pg/ml).

A sOX40L ELISA was constructed, optimized, and validated in-house. Maxisorb ELISA plates (NUNC, Roskilde, Denmark) were coated with a monoclonal mouse anti-OX40L antibody (MAB10541, R&D Systems, Minneapolis, MN, USA) diluted in PBS at a concentration of 4 ug/ml and blocked with ELISA blocking buffer (10% skimmed milk powder in PBS). Samples and standards (recombinant sOX40L, catalog number 310-28, Peprotech, Rocky Hill, NJ, USA) diluted 1:2 in PBS were added in duplicate and incubated overnight at 4°C. As above, all samples were pre-incubated with human, murine and bovine IgG in excess to ensure pre-aggregation of heterophilic antibodies. A biotinylated monoclonal mouse anti-OX40L (ANC10G1, Lifespan Biosciences, Seattle, WA, USA) was used as detection antibody at a concentration of 1 ug/ml in PBS. The minimal detectable concentration was 1.16 ng/ml for sOX40L.

Both ELISAs were spiked with variable concentrations of sOX40 and sOX40L, respectively. Recovery was >80% in all cases (data not shown). Furthermore, the samples were tested for the presence of sOX40/sOX40L dimers by adding the detection antibody from the sOX40 ELISA; this gave blank readings (data not shown). The cutoff of both ELISAs was calculated as the average value of blanks plus two SDs of the blanks. Values below the cutoffs were assigned the value of the cutoff.

### OX40 and OX40L flow cytometry

For this study, paired SFMC and PBMC samples from cRAs (n = 15) and PBMC samples from eRA (n = 5) and HVs (n = 10) were analyzed for the expression of OX40 and sOX40L. Surface staining was performed on unstimulated cells using mouse monoclonal antibodies against OX40 (clone MOPC-21, BD Biosciences, Heidelberg, Germany), OX40L (clone 159403, R&D Systems, Minneapolis, MN, USA), CD4 (clone RPA-T4, BD Biosciences), CD45RO (clone UCHL1, Beckman Coulter, Brea, CA, USA), CD14 (clone MϕP9, BD Biosciences), and CD19 (clone HD37, Dako Cytomation, Glostrup, Denmark). All antibodies were titrated to the optimal working concentrations. All samples were blocked for unspecific binding by preincubation with 10% heat-inactivated murine serum (in-house production). Gating of OX40 and OX40L was done using fluorescence minus-one (FMO) controls combined with the relevant isotype controls (FMO/iso) matched for species, IgG subtype, fluorochrome and concentration. All flow cytometric analyses were performed within 24 hours using the LSRFortessa with Diva 7 software (BD Biosciences) and FlowJo software (Tree Star Inc., Ashland, OR, USA). For gating strategy, please see Additional file 1: Figure S1.

### Statistics

Statistical analyses were performed using GraphPad Prism 6.0 for Mac (GraphPad Software, La Jolla, CA, USA). All data were analyzed by non-parametric statistics. Paired data were analyzed using the Wilcoxon signed-rank test. Non-paired data were analyzed by the Mann-Whitney *U*-test. Data were correlated using Spearman’s Rho. All data are expressed as median and IQR and 25th to 75th percentiles, unless otherwise specified. In all tests, the level of significance was a two-sided *P*-value <0.05.

## Results

### Soluble OX40 and sOX40L plasma levels are increased in eRA patients compared with HV

**Figure 1:**
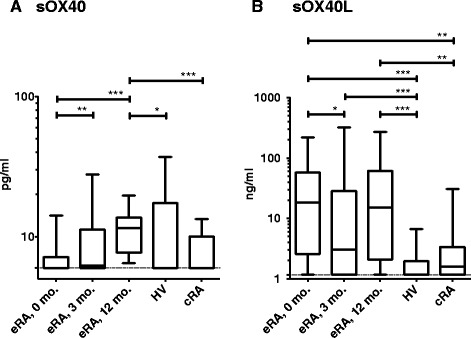
**Comparison of plasma sOX40 (A) and sOX40L (B) from patients with early rheumatoid arthritis (eRA) (n =76), chronic rheumatoid arthritis (cRA) (n = 15), and healthy volunteers (HV) (n = 34).** Plasma samples from patients with eRA were obtained at treatment initiation (0), and after 3 and 12 months of treatment. Patients with cRA had all had disease for more than 8 years. Paired data (within the eRA group) were analyzed by Wilcoxon signed-rank test, non-paired data were analyzed by Mann-Whitney *U*-test. Boxes represent median with interquartile range, and whiskers represent the 5th to 95th percentile: *P <0.05; **P <0.01; ***P <0.001. ELISA cutoff is indicated by the dotted line.

In the initial experiments, we wanted to determine the plasma level of sOX40 in eRA patients and to compare this level with the corresponding levels in cRA patients and HV. At the time of treatment initiation, eRA patients’ sOX40 levels were similar to those of cRA and HV. During the first treatment year sOX40 levels increased from 5.97 pg/ml (5.97 pg/ml to 7.14 pg/ml) at baseline to 11.47 pg/ml (7.69 pg/ml to 13.68 pg/ml) at 12 months (*P* <0.001), and sOX40 levels at 12 months were also significantly higher than those of cRA patients (5.97 pg/ml (5.97 pg/ml to 10.03 pg/ml) and HV (5.97 pg/ml (5.97 pg/ml to 17.34 pg/ml)) (both *P* <0.01, Figure 1A).

Plasma levels of sOX40L in eRA patients were significantly elevated throughout the first treatment year with a bimodal concentration curve where the highest levels were reached upon treatment initiation (18.30 ng/ml (2.59 ng/ml to 57.54 ng/ml)). This was followed by an 83% decrease after 3 months (3.06 ng/ml (1.16 ng/ml to 28.26 ng/ml), *P* <0.05) and a final increase to 15.10 ng/ml (2.09 ng/ml to 61.61 ng/ml) at 12 months (Figure 1B). The levels were significantly higher than in HV (1.16 ng/ml (1.16 ng/ml to 1.95 ng/ml) (*P* <0.0001, at all time points), and at baseline and 12 months when compared with cRA patients (1.59 ng/ml (1.16 ng/ml to 3.37 ng/ml) (both *P* <0.01)). Comparison of the two treatment groups (PLA, n = 37); ADA, n = 39) showed that there was no significant difference in their sOX40 and sOX40L plasma levels during the 12-month treatment period; thus, anti-TNF treatment seemed not to affect the shedding of the two proteins (data not shown).

### Soluble OX40L levels are connected to presence of anti-citrullinated protein antibodies (ACPA) and IgM-RF in eRA

We assessed whether sOX40L was associated with baseline antibody status, clinical disease parameters (DAS28CRP, clinical disease activity index (CDAI)) and CRP. We divided the eRA patients into IgM-RF and ACPA positive and negative at the time of treatment initiation. Plasma levels of sOX40L were strongly associated with the presence of IgM-RF and ACPA at baseline. In the IgM-RF-positive group sOX40L was 28.0 ng/ml (7.84 ng/ml to 75.48 ng/ml) versus 1.7 ng/ml (1.2 ng/ml to 3.4 ng/ml) in the IgM-RF-negative group (*P* <0.0001). Similarly, we observed sOX40L in the ACPA-positive group to have 23.2 ng/ml (3.6 ng/ml to 73.2 ng/ml) versus 2.7 ng/ml (1.2 ng/ml to 6.4 ng/ml) in the ACPA-negative group (*P* <0.001). This association with IgM-RF and ACPA was still present with sOX40L plasma levels measured at 3 months after treatment initiation (both *P* <0.01), but not after 12 months. Plasma levels of sOX40L in the IgM-RF-positive group was 22 ng/ml (2.68 ng/ml to 76.53 ng/ml) and 6.85 (1.82 ng/ml to 29.17 ng/ml) in the IgM-RF-negative group 12 months after treatment initiation, and no longer significant. Similarly, there was no difference in sOX40L between the ACPA-positive group (16.01 ng/ml (1.72 ng/ml to 60.0 ng/ml)) and the ACPA-negative group (12.0 ng/ml (2.79 ng/ml to 130 ng/ml)) after 12 months of treatment. Plasma levels of sOX40L did not at any time point correlate with disease activity parameters or CRP (Table 2).

**Table 2:** **Correlation between baseline plasma levels of sOX40L and sOX40 in early rheumatoid arthritis and clinical measurements**

	**sOX40L**	**sOX40**
DAS28 score, baseline	−0.039	0.152
DAS28 score, 12 months	0.027	0.209
HAQ score, baseline	0.031	0.189
HAQ score, 12 months	0.328**	−0.114
SDAI, baseline	−0.098	0.090
SDAI, 12 months	−0.039	−0.075
CDAI, baseline	−0.120	0.075
CDAI, 12 months	−0.113	0.079
IgM-RF, baseline	0.518**	0.003
ACPA, baseline	0.407**	0.005

Values shown are Spearman’s *rho* values, with *P <0.05, **P <0.01, ***P<0.001. CRP, C-reactive protein; DAS28, disease activity score,28 joints, CRP based; CDAI, clinical disease activity index; SDAI, simplified disease activity index; ACPA, anti-citrullinated protein antibodies; IgM-RF, IgM rheumatoid factor.

Systemic levels of sOX40 did not show any association with disease activity parameters, CRP, IgM-RF or ACPA (Table 2).

### The sOX40/sOX40L ratio is low in eRA and is associated with disease activity after adalimumab discontinuation

**Figure 2:**
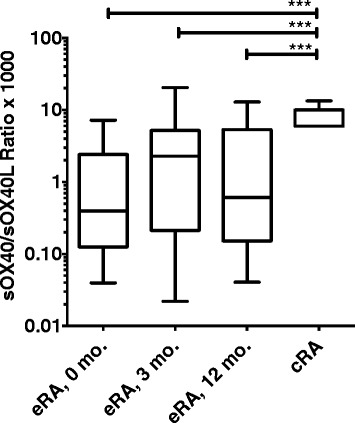
**Comparison of the ratio between sOX40 and sOX40L in early rheumatoid arthritis (eRA) and chronic rheumatoid arthritis (cRA).** The ratio was significantly lower in patients with eRA than in patients with cRA. Healthy volunteers are not included as many of the measurements for both sOX40 and sOX40L were below the level of detection. Paired data (within the eRA group) were analyzed by the Wilcoxon signed-rank test and non-paired data were analyzed by the Mann-Whitney *U*-test. Boxes represent median with interquartile range and whiskers represent the 5th to 95th percentiles. *P <0.05, ***P <0.001.

It has been suggested that like other members of the TNF superfamily, sOX40 and sOX40L function antagonistically to their membrane-bound forms. We therefore calculated the sOX40/sOX40L ratio to determine this axis in our cohort of eRA patients and correlated it with core disease parameters. Because sOX40 was measured in pg/ml and sOX40L was measured in ng/ml, the ratio was multiplied by a factor of 1,000. The sOX40/sOX40L ratio was significantly decreased in eRA compared with cRA throughout the study period (all *P* <0.001, Figure 2). We did not calculate a ratio for the HV, as many of the measurements for both sOX40 and sOX40L were below the detection limit. Adalimumab was discontinued after the first 12 month of treatment after which time patients underwent clinical examination at 3-months intervals for an additional year. Neither sOX40 nor sOX40L plasma levels were associated with change in disease activity (data not shown). However, patients with a high sOX40/sOX40L ratio at the time of adalimumab discontinuation had a significantly lower DAS28CRP after 3 months of follow up (*P* <0.05, data not shown). We did not observe the same correlation in the Placebo group (*P* = 0.9, data not shown). We also examined any association with disease flare and identified four patients with flare. They had a significantly decreased sOX40/sOX40L ratio compared with patients without flare (*P* <0.05, data not shown).

### OX40+ T cells and OX40L + B cells accumulate in the inflamed joints

**Figure 3:**
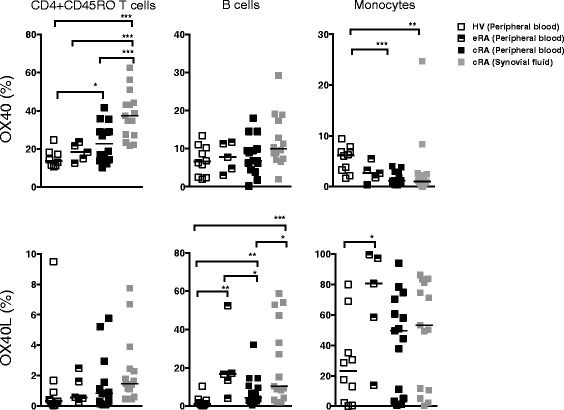
**Peripheral blood mononuclear cells (PBMC) and synovial fluid mononuclear cells (SFMC) from patients with chronic rheumatoid arthritis (cRA) (n = 15) and mononuclear cells from PBMC from** **early rheumatoid arthritis (eRA) (n =5) and healthy volunteers (HV) (n = 10) were examined by flow cytometry for expression of CD4, CD14, CD19, CD45RO, OX40, and OX40L.** Closed squares = peripheral blood from patients with cRA; semi-solid squares = peripheral blood from patients with eRA; gray squares = synovial fluid from patients with cRA; white squares = peripheral blood from healthy volunteers. We observed an increased percentage of OX40- and OX40L-expressing cells in the synovial fluid, with CD4 + CD45RO + T-cells being more positive for OX40 and CD19+ B cells and monocytes being more positive for OX40L. Paired data (PBMC and SFMC) were analyzed by Wilcoxon signed-rank test, non-paired data were analyzed by the Mann-Whitney *U*-test. Solid lines represent median with interquartile range and whiskers represent the 5th to 95th percentiles. *P <0.05 and **P <0.01, ***P <0.001.

We investigated which cells express the membrane-bound isoforms. PBMC from eRA (n = 5), and paired SFMC and PBMC from cRA (n = 15) were examined for expression of OX40 and OX40L by T cells, B cells, and monocytes. As expected, OX40 was mainly expressed by CD4 + CD45RO + T cells in the inflamed joint compared with peripheral blood (Figure 3). The highest percent of OX40-expressing cells, was seen on CD4 + CD45RO + T cells of cRA SFMC (37.5% (27.1% to 44.2%)) compared with cRA PBMC (22.9% (14.3% to 30.7%)), eRA PBMC (18.3% (13.8% to 22.65%)) and HV PBMC (13.7% (11.2 to 18.1%)) (all *P* <0.001). OX40+ cells were significantly higher on CD4 + CD45RO + T cells of cRA PBMC compared with HV PBMCs (*P* <0.05). OX40+ B cells and monocytes were only present in low numbers.

OX40L was primarily expressed by B cells and monocytes, which was consistent with the fact that OX40L is expressed mostly on antigen-presenting cells. OX40L expressing B cells were significantly increased in SFMC from cRA patients (10.3% (4.3% to 47.3%)) as compared with cRA PBMC (4.5% (2.7% to 10.6%)) and HV PBMC (1.2% (0.5% to 3.2%)) (*P* <0.05 and *P* <0.001). The percentage of OX40L expressing B cells from cRA PBMC was significantly increased compared with HV (*P* <0.01). The percentage of B cells expressing OX40L in PBMCs from eRA (16.7% (8.7% to 34.8%)) was also significantly increased compared with both cRA and HV (*P* <0.05 and *P* <0.01, respectively). Generally, a higher percentage of monocytes were OX40L + compared with B cells. A higher percentage of monocytes from eRA PBMCs were OX40L + (80.7% (36.1% to 98.5%)) compared with monocytes from HV PBMCs (23.1% (2.0% to 43.6%)) (*P* <0.05). As mentioned above, only 5 eRA PBMC samples were analyzed, and the results should therefore be interpreted with caution.

## Discussion

The generation of immunological memory is a hallmark of the adaptive immune system. OX40 and OX40L are central players in providing co-stimulatory signals to activated effector CD4+ T cells and antigen-presenting cells during this process [7,14].

In the present study, we present the first data on the presence of sOX40 and sOX40L in patients with early treatment naïve RA and the association between the soluble forms of these TNFR superfamily receptors and clinical disease parameters. We demonstrate that patients with early RA have altered systemic levels of sOX40 and sOX40L compared to HV and cRA, and that the highest levels are seen in ACPA- and IgM-RF-positive eRA. We further had indications that the balance between sOX40 and sOX40L is associated with a risk of increase in DAS28CRP after adalimumab discontinuation.

The elevated sOX40L levels and the gradually rising sOX40 levels during the first year after diagnosis point to ongoing activity in the adaptive memory system throughout this period. By contrast, levels of sOX40 and sOX40L were almost normalized in cRA, although the percentage of cells expressing OX40 and OX40L was much alike in cRA and eRA. Little is known about how the shedding of OX40 and OX40L occurs, but the process is well described for the TNF superfamily member TNFα, and the process is known to be tightly regulated [19]. Our results suggest that the mechanisms involved in shedding of OX40 and OX40L in cRA are changed as compared to eRA.

Even though the eRA patients all experienced clinical benefit from treatment and more than 50% achieved European League against Rheumatism (EULAR) remission [20], this was not reflected in the sOX40 and the sOX40L levels. This could either mean that the OX40 system is upstream of TNFα-regulated processes or that they are uncoupled processes. The latter is more likely, as signals through the OX40 system are known to be necessary for stable antibody response formation, in this case rheumatoid factor and ACPA antibodies, and that patients undergoing anti-TNF therapy are able to mount adequate vaccine responses [21,22].

The potential of the OX40 system in generating immunological memory is particularly interesting in RA. Like other studies, the OPERA study showed that RA patients who have achieved remission can discontinue anti-TNF therapy if intensively treated in the early phase of disease [23,24]. This suggests the presence of a window of opportunity in the early phase of RA. Conversely, a subgroup of patients has a relatively poor prognosis. To maximize the treatment effect, it is evidently of great interest to separate these two groups. In our study, patients with high sOX40L plasma levels at baseline and after 3 months were significantly more likely to be positive for ACPA and IgM-RF at baseline. At later time points this correlation was not significant. This was not due to seroconversion, but because the levels of sOX40L changed from baseline to 12 months after treatment initiation in the individual patients, even though the median value was relatively unchanged. The strong association between sOX40L and autoantibodies, supports the role of the OX40-system in generating adaptive immunity. This is in line with a recent study, showing that the OX40-system is central to ensure optimal priming of B-cell to generate immunity via CD4 T-cells [25]. OX40 is expressed by activated effector T cells and OX40L by B cells. In our study, we found both of these molecules to be upregulated during the first year after diagnosis. This create an optimal environment for generation of effector T-cells to facilitate initiation of antibody formation and maturation of long-lived plasma cells secreting antibodies [25,26].

The importance of the imbalance between sOX40 and sOX40L is supported by the association between the sOX40/sOX40L ratio and disease activity after adalimumab discontinuation. A low ratio was significantly associated with a higher DAS28CRP score after 3 months. However, defined flare (DAS28CRP >1.2 if DAS28CRP <3.2 or >0.6 if DAS28CRP ≥3.2 [17]) was only observed in four out of the 39 RA patients that discontinued adalimumab, and the results should therefore be analyzed cautiously, until confirmed in a larger cohort. The low flare rate is most likely due to the short follow-up intervals combined with an aggressive regime of intra-articular triamcinolone prescribed by the OPERA protocol. The possible link between the sOX40/sOX40L ratio and the risk of flare is, of course, of clinical interest as these findings may help predict flare in RA patients, which is currently not possible. In combination with the accumulation of cells expressing OX40 and OX40L in the inflamed joints, this underscores the potentially central role of the OX40 system in perpetuating the autoimmune process in RA. These findings are supported by studies of collagen II-induced arthritis in mice where administration of anti-OX40L mAb ameliorated the disease [17].

We hypothesize that the modulation of RA disease processes through the mechanisms exercised by the sOX40 and sOX40L molecules could take two forms. Soluble OX40 possibly works as a decoy receptor and blocks sOX40L or membrane-bound OX40L from binding to membrane-bound OX40. As a result, it inhibits proliferation and survival signals for the cells. Previous studies have shown that sOX40 mimics the anti-inflammatory function of inducible T-regulatory (Treg) cells and thereby suppresses the autoreactive T cells [8]. By contrast sOX40L is more likely to act as a survival factor in the continuous activation of T cells and B cells, making rheumatoid factor or ACPA antibodies synthesis possible. This gives rise to dysregulation of the generation of immunological memory and is in accordance with our findings in the sense that eRA has low sOX40/OX40L ratio, reflecting the balance of a more pro-inflammatory cellular environment in this disease.

## Conclusions

In summary, we found that sOX40 and sOX40L levels were changed in eRA and that sOX40L was associated with IgM-RF and ACPA. Furthermore, sOX40/sOX40L ratio was associated with increase in DAS28CRP after adalimumab discontinuation. In addition, the number of cells expressing membrane-bound OX40 and OX40L was increased in RA. Overall, our findings suggest that the OX40 system is important for the formation of key autoantibodies in RA and possibly associated with future disease activities.

## Additional file

Additional file 1: Figure S1. Representative plots showing gating on OX40- and OX40L-expressing cells. Gates were set using fluorescence minus one (FMO) controls combined with a matched isotype antibody, thus, correcting for both uncompensated spectral overlap and unspecific binding. T cells were gated as CD4 + CD45RO+. B cells were gated as CD19+. Monocytes were gated using CD14. All cells were blocked using 10% heat-inactivated murine serum to avoid unspecific binding.

## Abbreviations

ACPA: anti citrullinated peptide antibody
ADA: patient group receiving adalimumab and methotrexate
APC: antigen-presenting cell
BT: biotinyl thyramide
CDAI: clinical disease activity index
cRA: chronic rheumatoid arthritis
CRP: C-reactive protein
DAS28: disease activity in 28 joints
DAS28CRP: C-reactive protein-based disease activity in 28 joints
DMARD: disease-modifying anti-rheumatic drugs
EDTA: ethylenediaminetetraacetic acid
ELISA: enzyme-linked immunosorbent assay
eRA: early rheumatoid arthritis
FMO: fluorescence minus one
FSC: forward scatter
HAQ: health assessment questionnaire
HV: healthy volunteers
IgG: immunoglobulin G
IgM-RF: IgM rheumatoid factor
INART: inflammation in arthritis
IQR: interquartile range
MRI: magnetic resonance imaging
MTX: methotrexate
NF-κB: nuclear factor kappa-light-chain-enhancer of activated B cells
OD: optical density
OPERA: optimized treatment algorithm in early rheumatoid arthritis
PBMC: peripheral blood mononuclear cells
PBS: phosphate-buffered saline
PLA: patient group receiving placebo and methotrexate
RA: rheumatoid arthritis
RCT: randomized controlled trial
SDAI: simple disease activity index
SFMC: synovial fluid mononuclear cells
SSC: side scatter
TMB: tetramethylbenzidine
TNF: tumor necrosis factor
TNFα: tumor necrosis factor alpha
T-reg: T regulatory cell
