# Supplementary figures and images for: Soluble OX40L is associated with presence of autoantibodies in early rheumatoid arthritis
Source: Arthritis Res Ther. 2014 Oct 30;16(5):474. doi: 10.1186/s13075-014-0474-4 (PMC4230735; doi:10.1186/s13075-014-0474-4)

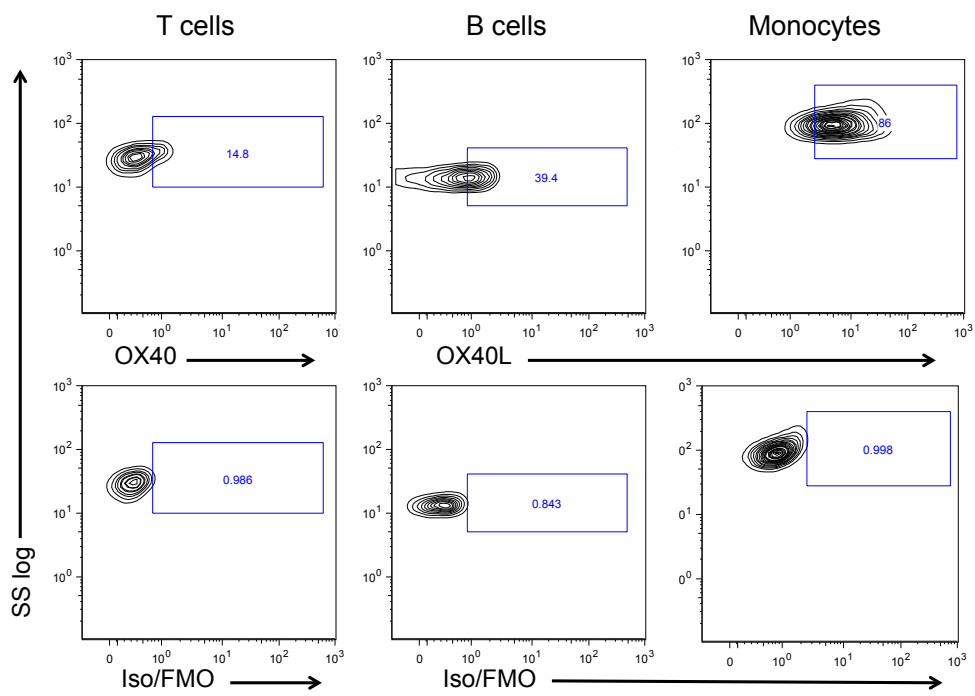

Supplement: Additional file 1: Figure S1. — Representative plots showing gating on OX40- and OX40L-expressing cells. Gates were set using fluorescence minus one (FMO) controls combined with a matched isotype antibody, thus, correcting for both uncompensated spectral overlap and unspecific binding. T cells were gated as CD4 + CD45RO+. B cells were gated as CD19+. Monocytes were gated using CD14. All cells were blocked using 10% heat-inactivated murine serum to avoid unspecific binding. [file 13075_2014_474_MOESM1_ESM.pdf]
